# Integrated miRNA-seq and RNA-seq analysis reveals stage-specific miRNA-mRNA regulatory networks in *Populus yunnanensis* under salt stress

**DOI:** 10.1186/s12864-026-12882-w

**Published:** 2026-04-27

**Authors:** Yuxia Zhang, Yude Kang, Jiajun Zhuo, Xiaojiao Liu, Lincui Shi, Aizhong Liu, Ping Li

**Affiliations:** 1 Key Laboratory for Forest Resource Conservation and Utilization in the Southwest Mountains of China (Ministry of Education), College of Forestry, Southwest Forestry University, Kunming, China; 2 Key Laboratory for Conservation and Utilization of in forest Resource of Yunnan, Southwest Forestry University, Kunming, China

**Keywords:** *Populus yunnanensis*, Salt stress, MicroRNAs, Transcriptome, Regulatory network

## Abstract

**Background:**

Soil salinization is a major abiotic stress that severely constrains global forestry productivity. Plants deploy sophisticated gene regulatory networks for adaptation, in which microRNAs (miRNAs) have emerged as crucial post-transcriptional modulators. However, a comprehensive understanding of the dynamic and phase-specific roles of miRNAs in the salt stress response of perennial woody plants remains limited.

**Results:**

We performed an integrated miRNA-seq and RNA-seq analysis on *Populus yunnanensis* leaves across four treatment points: control (CK), short-term salt stress (T1) and long-term salt stress (T4), and recovery after stress (TR). Small RNA sequencing identified 571 miRNAs, including 339 known and 232 novel candidates, with expression dynamics highly sensitive to stress phases. Differential expression analysis revealed stage-specific miRNA repertoires across five biologically defined phases: Early Response (ER, T1vsCK; 6 DEMs), Long-term Adaptation (LA, T4vsCK; 17 DEMs), Recovered State (RS, TRvsCK; 17 DEMs), Stress Progression (SP, T4vsT1; 15 DEMs), and Recovery Process (RP, union of TRvsT1 and TRvsT4; 18 DEMs). Notably, ptc-miR6462 and ptc-miR6476 were unique to ER; ptc-miR169 was specific to SP; ptc-miR6457 was specific to RP; ptc-miR477 was unique to RS; while ptc-miR395 family members were active across all stages except ER. Concurrent transcriptomics unveiled extensive, duration-dependent transcriptional reprogramming, with DEGs increasing from 603 in ER to 3,027 in LA, and 2,239 DEGs persisting in RS. KEGG enrichment highlighted the central role of metabolic pathways across all stages, with phase-specific activation of signaling pathways (MAPK, plant hormone transduction) in LA and RS, membrane remodeling pathways (alpha-Linolenic acid metabolism) in RP, and sustained metabolic adjustments (cysteine and methionine metabolism, secondary metabolite biosynthesis) in RS. Integrated miRNA-mRNA network analysis constructed core regulatory circuits underpinning stage-specific adaptation, including ptc-miR395-APS1 (ATP sulfurylase 1) for antioxidant synthesis, ptc-miR319-MYB for growth-defense balance during recovery, and novel circuits involving ptc-miR6457b-MazG and ptc-miR6476-GINS, suggesting roles in nucleotide homeostasis and DNA replication protection.

**Conclusions:**

Our study delineates a complex, phase-specific post-transcriptional regulatory network that orchestrates the salt stress adaptation of *P. yunnanensis*. By distinguishing five distinct phases-early response, long-term adaptation, stress progression, recovery process, and recovered state-we demonstrate that miRNAs function as precise temporal tuners, sequentially regulating distinct biological processes: from initial signal perception and DNA protection (ER), through sustained metabolic adaptation and antioxidant defense (LA, SP), to active recovery mechanisms (RP) and the establishment of stress memory (RS). These findings provide novel insights into the molecular basis of salt tolerance in trees and furnish valuable genetic resources for breeding stress-resilient forest varieties.

**Supplementary Information:**

The online version contains supplementary material available at 10.1186/s12864-026-12882-w.

## Introduction

Soil salinization is one of the most severe environmental stresses, posing an expanding threat to global agriculture and forestry. It is estimated that over 50% of arable land may be affected by 2050 [1]. For plants, high salinity induces a complex suite of physiological injuries, including ionic toxicity, osmotic stress, and secondary oxidative damage, which collectively inhibit growth and development [2]. To survive, plants have evolved sophisticated adaptive mechanisms spanning physiological, biochemical, and molecular levels. These include the activation of hormone signaling pathways, the synthesis of osmoprotectants, enhanced reactive oxygen species (ROS) scavenging systems, and the well-conserved Salt Overly Sensitive (SOS) pathway for ion homeostasis [3]. The regulation of these processes involves a multi-layered gene regulatory network, encompassing transcription factors, epigenetic modifications, and critically, post-transcriptional regulators [4].

Among the key post-transcriptional regulators are microRNAs (miRNAs), a class of ~ 21 nucleotide endogenous non-coding RNAs. miRNAs function by guiding the cleavage or translational inhibition of target mRNAs through sequence complementarity, thereby acting as high-level switches in gene regulatory networks [5, 6]. They are deeply involved in nearly all aspects of plant biology, from growth and development to responses to abiotic stresses. The development of high-throughput sequencing (HTS) technologies has been instrumental in uncovering numerous stress-responsive miRNAs [7]. For instance, specific miRNAs such as miR396, miR169, miR395, and miR398 have been implicated in salt tolerance in plants like rice [8], creeping bentgrass [9], *Arabidopsis thaliana* [10], and rice [11]. These studies collectively demonstrate that miRNAs confer stress adaptation by targeting a wide spectrum of genes, including those encoding transcription factors, hormone signaling components, and metabolic enzymes [12, 13].

Critically, miRNA-mediated regulation is highly dynamic and sensitive to stress intensity, duration, and developmental stage. For example, in sugarcane, different NaCl concentrations and treatment times lead to distinct miRNA expression profiles, revealing the sensitivity and complexity of miRNA functions [14]. Similarly, in the euhalophyte *Salicornia europaea* and the energy crop *Arundo donax* L., transcriptomic analysis reveal that gene expression undergoes deeper and more complex reprogramming under long-term salt stress compared to short-term exposure [12, 15]. This temporal dimension is essential, as the plant stress response unfolds in distinct physiological phases: initial shock, acclimation, and potential recovery or priming for future stress. Research in rice shows that plants subjected to salt stress at different growth stages exhibit different transcriptional and physiological responses to secondary stress events, hinting at the establishment of stress memory [16]. These findings collectively suggest that the miRNA repertoire and their target networks are not static but are deployed in a phased, stage-specific manner to manage immediate damage, establish new homeostasis, and coordinate recovery or preparedness.

Despite these advances, significant knowledge gaps remain, particularly in perennial woody plants. Firstly, most discoveries have been made in herbaceous models, and the miRNA repertoire and its functional conservation or divergence in trees are poorly explored. Secondly, and more critically, the majority of studies offer only a static snapshot of miRNA expression at a single time point. There is a pressing need to capture the dynamic reprogramming of miRNA-mRNA regulatory networks across the entire trajectory of stress response, including a recovery phase, to understand how trees manage resource allocation and potentially develop stress memory [16, 17]. We hypothesize that salt stress adaptation in woody plants is orchestrated by a phased and stage-specific miRNA-mRNA regulatory network, with distinct modules governing early signal perception, sustained metabolic adaptation, active recovery, and the establishment of stress memory.

*Populus yunnanensis*, an ecologically and economically important forest tree native to southwestern China [18], provides an excellent system to test this hypothesis. In this study, we employed an integrated multi-omics approach, conducting simultaneous miRNA-seq and RNA-seq analyses on *P. yunnanensis* leaves subjected to short-term salt stress, long-term salt stress, and a subsequent recovery period. Our objectives were threefold: (1) comprehensively identify conserved and novel miRNAs and characterize their dynamic expression patterns across five biologically defined phases-Early Response, Long-term Adaptation, Stress Progression, Recovery Process, and Recovered State; (2) analyze the global transcriptomic reprogramming and functional enrichment across these critical phases; (3) integrate the miRNA and mRNA data to construct phase-specific regulatory networks, thereby elucidating the key post-transcriptional circuits that underpin salt adaptation in a woody plant. Our findings provide not only fundamental insights into the temporal regulatory logic of tree stress biology but also valuable genetic resources for the molecular breeding of salt-tolerant forest varieties.

## Materials and methods

### Plant materials and salt stress treatment

The plant material used in this study was *Populus yunnanensis*, a species native to southwestern China. The plants were propagated from cuttings obtained from a long-term cultivated population maintained in the greenhouse of Southwest Forestry University. The species identity was confirmed based on morphological characteristics and comparison with taxonomic descriptions of *P. yunnanensis* [18, 19]. The plant cuttings were obtained from one-year-old branches and cultivated in a greenhouse at Southwest Forestry University for two months under a 16/8 h light/dark cycle at 25/18°C. Plants were grown in a mixed substrate of humus, quartz sand, and perlite (3:1:1, v/v/v). Uniform two-month-old plants were selected and acclimated in a hydroponic system with 1/4 Murashige and Skoog (MS) nutrient solution for two weeks prior to treatments.

The salt stress experiment included the following treatments: (i) Control (CK): continuous growth in 1/4 MS solution; (ii) Short-term salt stress (T1): exposure to 25 mM NaCl for 2 days; (iii) Long-term salt stress (T4): following T1 and a 3-day recovery in fresh 1/4 MS solution, plants underwent a sequential treatment: 50 mM NaCl for 2 days, recovery in fresh 1/4 MS solution for 3 days, followed by two cycles of 75 mM NaCl for 2 days each, interspersed with a 3-day recovery in fresh 1/4 MS solution as adapted from our previous study [20]; (iv) Recovery after stress (TR): after the T4 treatment, plants were returned to fresh 1/4 MS solution for 21 days. Fully expanded leaves from the middle part of the stem (the 4th to 6th leaves from the apex) from each treatment group were collected, immediately frozen in liquid nitrogen, and stored at -80 °C for subsequent RNA extraction and high-throughput sequencing. Three biological replicates were included per treatment.

### Small RNAs sequencing and data analysis

Total RNA was extracted from leaf samples using TRIzol reagent (Invitrogen, USA) according to the manufacturer’s instructions. Small RNA library construction and 50 bp single-end sequencing were conducted by Novogene Bioinformatics Technology Co., Ltd. (Beijing, China). Twelve libraries (CK, T1, T4, TR; n = 3 replicates) were prepared using the NEB Next^®^ Multiplex Small RNA Library Prep Set for Illumina^®^ (NEB, USA). Briefly, 3’ and 5’ adapters were ligated to small RNAs, followed by reverse transcription and PCR amplification. Libraries with insert sizes of 18–40 bp were selected and subjected to 50 bp single-end sequencing on an Illumina platform.

Raw sequencing reads in FASTQ format [21] were initially subjected to quality control using FastQC (version 0.11.9). Adapters, reads containing poly-N, low-quality reads (Q-score < 20), and reads shorter than 18 nt or longer than 30 nt were removed using TrimGalore (version 0.6.4) to obtain high-quality clean reads. Quality metrics, including Q20, Q30, and GC content, were calculated for the clean data. A total of 127 million raw reads were generated, yielding 97 million clean reads after quality control, with retention rates ranging from 62.58% to 83.82% across libraries (Additional file 1: Table S1). The length distribution of both unique and total reads was analyzed within the 18–30 nt range. Unique reads were generated by collapsing identical sequences using the miRDeep2 (version 0.1.3) (mapper.pl) script [22]. These unique reads were then aligned to the *P. yunnanensis* reference genome using Bowtie (version 1.2.3) allowing no mismatches. The mapped sequences were subsequently annotated by alignment against the miRBase (release 22, https://www.mirbase.org/) and Rfam (release 14.4, https://ftp.ebi.ac.uk/pub/databases/Rfam/) databases, as well as genomic features (repeat sequences, exons, and introns) of *P. yunnanensis*, to categorize small RNAs into known miRNAs, rRNAs, tRNAs, snRNAs, snoRNAs, other non-coding RNAs, repeats, exons, introns, and unknown sequences. The miRBase database of *Populus trichocarpa* was set as the main reference of *P. yunnanensis* miRNAs. Sequences classified as unknown and originating from introns were extracted for novel miRNA prediction using the miRDeep2 core module with default parameters.

The expression levels of small RNAs were quantified based on transcripts per million (TPM). Differential expression analysis of miRNAs was performed using the DESeq2 package (version 1.32.0) in R (version 4.4.2), with significance thresholds set at |log_2_(Fold Change)| ≥ 1 and *p-*value < 0.05. Following standard miRBase nomenclature, all identified known miRNAs are designated with the species prefix “ptc-” (e.g., ptc-miR395a), which indicates their origin in the *P. trichocarpa* reference database. This prefix is retained throughout the manuscript to maintain consistency with the miRBase annotation, although the miRNAs were identified in *P. yunnanensis*.

### Transcriptome sequencing and analysis

Strand-specific transcriptome library construction and 150 bp paired-end sequencing were performed by Novogene Bioinformatics Technology Co., Ltd. (Beijing, China). In brief, ribosomal RNA was depleted from total RNA using the Ribo-Zero rRNA Removal Kit (Illumina, USA), and the resulting fragments were used for cDNA synthesis. The synthesized double-stranded cDNA underwent terminal repair, A-tailing, adapter ligation, and PCR amplification to construct the final library. After quality control, the libraries were sequenced on an Illumina NovaSeq 6000 platform, generating approximately 78–84 million clean reads per library (Additional file 1: Table S2).

Raw reads quality was assessed using fastp (version 0.20.0). Clean reads were obtained by removing reads containing adapter contamination, low-quality nucleotides (Q-score < 20), and unrecognized nucleotides (N). Clean reads were aligned to the *P. yunnanensis* genome using HISAT2 (version 2.1.0), and sorted BAM files were generated with Samtools (version 1.10). Read count matrix was generated using the featureCounts from the Rsubread package (version 2.6.4) [23]. Differential gene expression analysis was conducted with DESeq2 (version 1.32.0), with significance thresholds set at adjusted *p*-value < 0.05 and |log_2_(FC)| ≥ 1. Gene Ontology (GO) enrichment and Kyoto Encyclopedia of Genes and Genomes (KEGG) analysis were performed using the Omicshare website tools (www.omicshare.com/tools) with default parameters.

### Integrated analysis of miRNA-mRNA regulatory networks

The targeting relationships between differentially expressed miRNAs (DEMs) and differentially expressed genes (DEGs) were predicted using both psRobot (version 1.2, http://omicslab.genetics.ac.cn/psRobot/) and TargetFinder (version 1.6, http://targetfinder.org/). For psRobot, we used the default parameters with an expectation value ≤ 2.5 as the threshold for high-confidence predictions. The expectation value represents the probability of finding a match by chance, with lower values indicating more reliable predictions. For TargetFinder, predictions were performed using the default scoring system, and we retained interactions with a score > 4, which indicates higher probability of true targeting based on sequence complementarity, target site accessibility, and evolutionary conservation.

Only miRNA-mRNA pairs identified by both tools with the above thresholds were considered high-confidence and retained for subsequent analysis. To identify potential functional regulatory relationships, we further filtered these pairs based on expression patterns: (1) both miRNA and target gene were differentially expressed in the same phase; (2) their expression patterns were anti-correlated (miRNA up-regulated with target down-regulated, or vice versa); and (3) the Pearson correlation coefficient calculated from TPM values across all samples was < -0.8 with *p*-value < 0.05. These high-confidence, anti-correlated pairs were used to construct stage-specific regulatory networks for five biologically defined phases: Early Response (ER, T1vsCK), Long-term Adaptation (LA, T4vsCK), Recovered State (RS, TRvsCK), Stress Progression (SP, T4vsT1), and Recovery Process (RP, union of TRvsT1 and TRvsT4). Network visualization was performed using Cytoscape (version 3.10.2).

### RNA isolation and qRT-PCR validation

Three miRNA-mRNA pairs were randomly selected for experimental validation by quantitative reverse transcription polymerase chain reaction (qRT-PCR). For each treatment group, three biological replicates were analyzed.

For miRNA analysis, total RNA was extracted using the miRcute miRNA Isolation Kit (TIANGEN, China). cDNA was synthesized from 1 µg of total RNA using the miRcute Plus miRNA First-Strand cDNA Kit (TIANGEN, China). qRT-PCR reactions were performed in a 20 µl system containing 2 µl cDNA, 0.25 µM of each primer, and 10 µl of 2× miRcute Plus miRNA PreMix (SYBR Green) on a Bio-Rad CFX96 system. The thermocycling conditions were: 95 °C for 15 min; 5 cycles of 94 °C for 20 s, 65 °C for 30 s, 72 °C for 34 s; followed by 45 cycles of 94 °C for 20 s, 60 °C for 34 s. U6 snRNA was used as the endogenous control [24].

For mRNA analysis, total RNA was extracted using the RNAprep Pure Plant Plus Kit (TIANGEN, China). After genomic DNA removal, 4 µg of total RNA was reverse-transcribed using the HiScript^®^ III 1st Strand cDNA Synthesis Kit (Vazyme, China). qRT-PCR was performed on a Bio-Rad CFX96 system using 2× Universal SYBR qPCR Master Mix (YUNBIO, China) according to the manufacturer’s instructions. To select an appropriate endogenous control for mRNA normalization, we evaluated the expression stability of several candidate reference genes across all samples (CK, T1, T4, TR) using our transcriptome dataset. Based on this analysis, the gene *Poyun22323*, which encodes a LETM1 and EF-hand domain-containing protein and exhibited the most stable expression across all conditions, was chosen as the reference gene for mRNA qRT-PCR validation. The 2⁻ΔΔCt method was used for relative quantification [25]. All reactions were run with three technical replicates. Gene-specific primers are listed in Additional file 1: Table S3.

## Results

### miRNAs involved in the salt stress response of *P*. *yunnanensis*

**Fig. 1:**
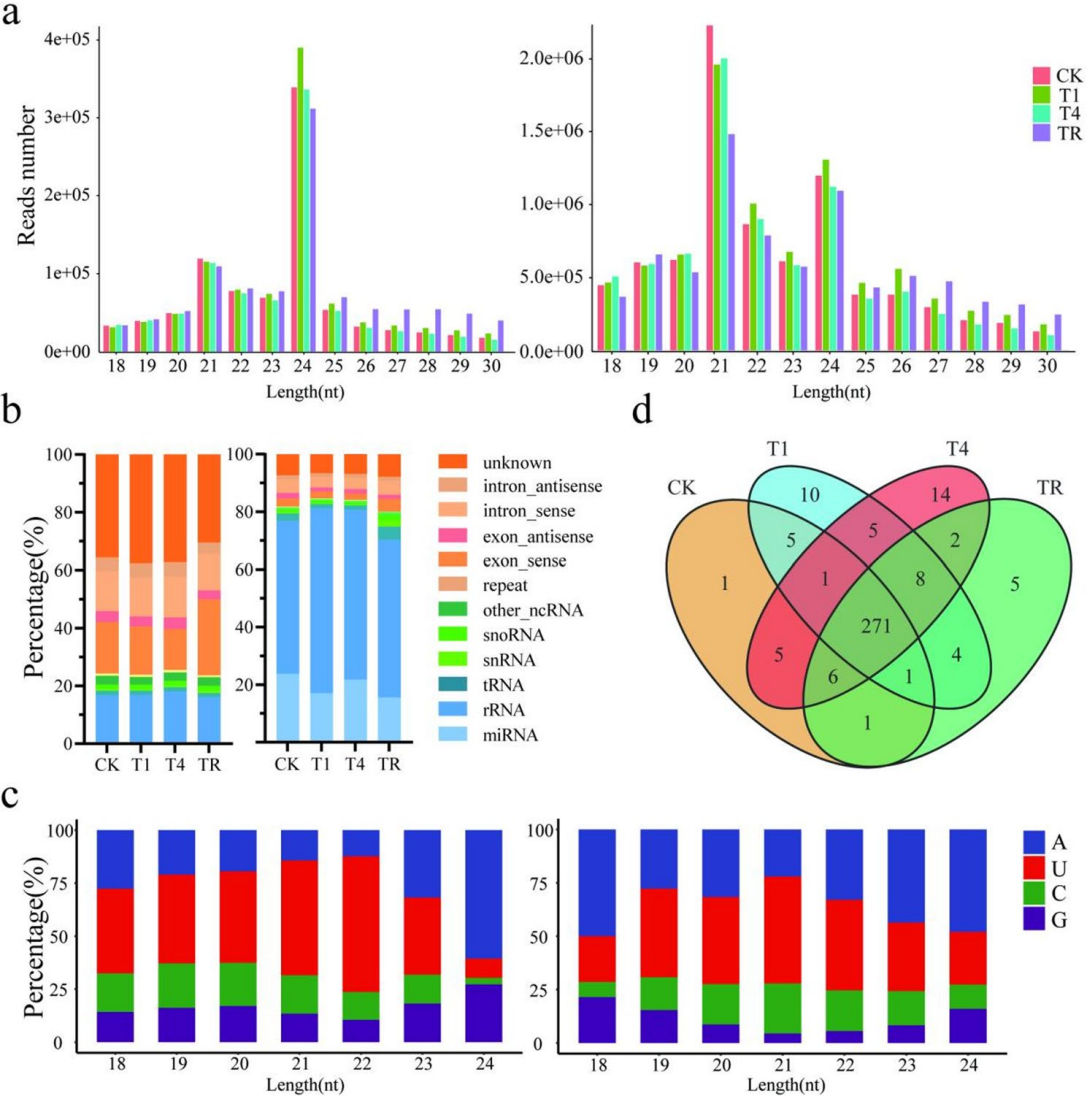
Characterization of small RNA sequences from 12 libraries of *P. yunnanensis* leaf tissues.** a** Length distribution of unique (left) and total (right) sRNA reads. **b** Annotation of unique and total clean reads based on alignment to the *P. yunnanensis* genome, Rfam, miRBase, and genomic features. Unknown, unmapped sequences; snoRNA, small nucleolar RNA; snRNA, small nuclear ribonucleic acid; tRNA, transfer RNA; rRNA, ribosomal RNA; miRNA, microRNA. **c** First nucleotide bias of known (left) and novel (right) miRNA unique reads. **d** Venn diagram of miRNAs identified across salt treatment stages. CK, control; T1, short-term salt stress; T4, long-term salt stress; TR, recovery after stress

We constructed 12 small RNA libraries from leaves of *P*. *yunnanensis* under four conditions: control (CK), short-term salt stress (T1), long-term salt stress (T4), and recovery after stress (TR), with three biological replicates per condition. A total of 127 million raw reads were generated, yielding 97 million clean reads after quality control, with retention rates ranging from 62.58% to 83.82% (Additional file 1: Table S1). The length distribution of both unique and total reads ranged from 18 to 30 nt. Among unique reads, 24 nt sRNAs were the most abundant, followed by 21 nt species. In contrast, among total reads, 21 nt sRNAs predominated, followed by 24 nt sRNAs (Fig. 1a). This discrepancy suggests a greater diversity of 24 nt siRNAs, often associated with heterochromatin and repetitive sequences, while 21 nt miRNAs, though encoded by fewer loci, are expressed at much higher levels due to their potent and specific regulatory roles.

The sRNAs were categorized into miRNA, rRNA, tRNA, snRNA, snoRNA, repeat, exon, intron, other non-coding RNAs, and unknown sequences (Fig. 1b). Known miRNAs predominantly started with “U” at their 5’ end, whereas 24 nt miRNAs mostly began with “A”. Novel miRNAs also showed a “U” bias, except for 18, 23, and 24 nt miRNAs, which started mainly with “A” (Fig. 1c). In total, we identified 339 known miRNAs across the four treatment points, including 164 known miRNAs belonging to 36 families, while the remaining 175 miRNAs were classified as unknown families and labeled as “NA” (Fig. 1d, Additional file 1: Table S4). Additionally, 232 novel miRNAs were predicted, with their genomic locations, precursor and mature sequences provided (Additional file 1: Table S5). The numbers of miRNAs identified in each treatment are summarized in Additional file 2: Fig. S1a.

The abundance of these miRNAs changed dynamically across treatment points. Specifically, 291 miRNAs were expressed in CK, increasing to 305 and 312 during T1 and T4, respectively, then decreasing to 298 after TR (Fig. 1d, Additional file 1: Table S6).

### Differential response of miRNAs across salt stress treatments in *P. yunnanensis*

**Fig. 2:**
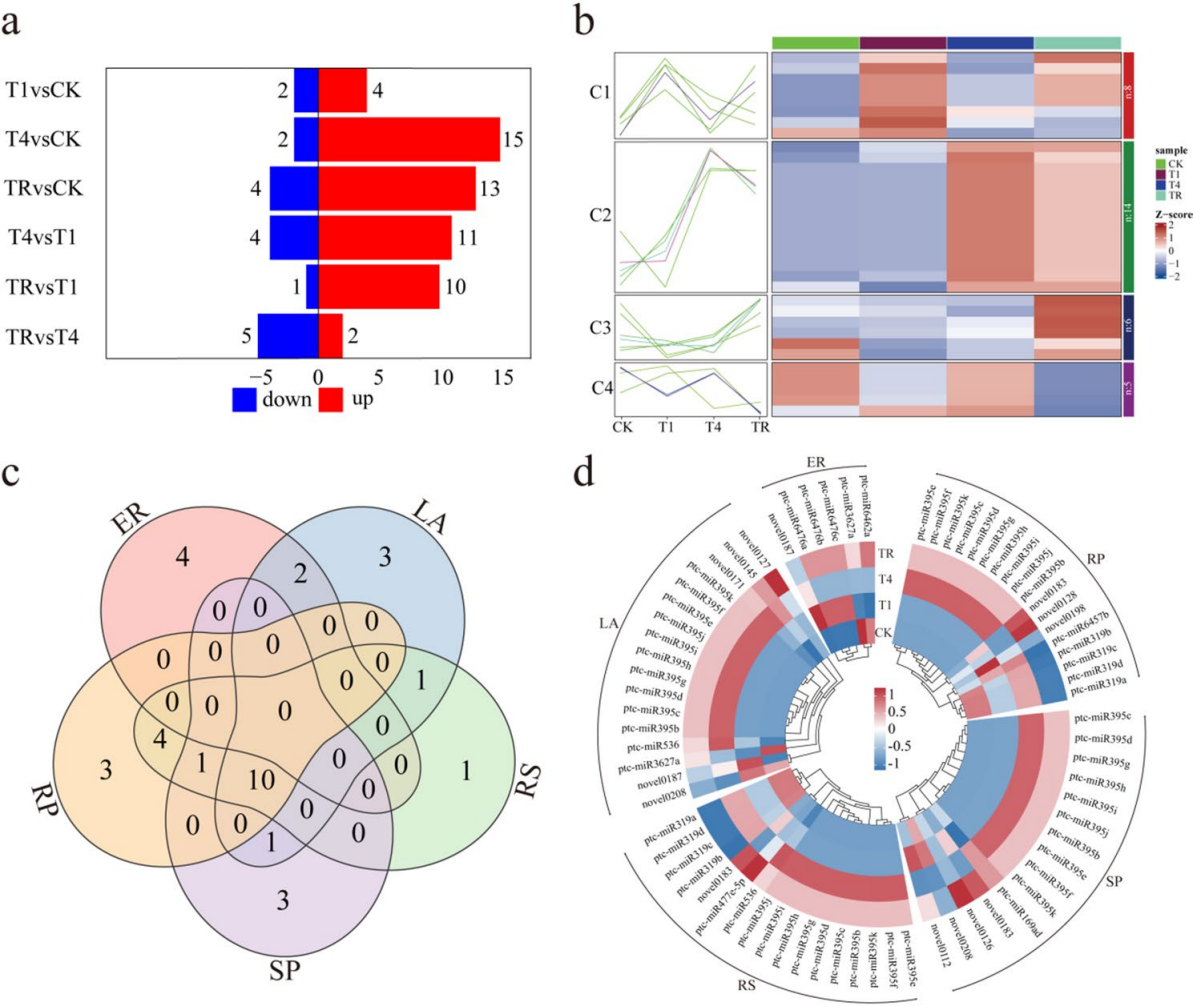
Dynamic response of miRNAs to salt stress in *P. yunnanensis*. **a** Bar plot showing the number of up- and down-regulated differentially expressed miRNAs (DEMs) in the six indicated pairwise comparisons. (|log_2_(Fold Change)| ≥1, *p*-value < 0.05). **b** Heatmap of hierarchical clustering of all DEMs across the six comparisons. Rows represent miRNAs and columns represent four treatment points. The color scale represents Z-score normalized expression levels, revealing four major clusters of expression trends. **c** Venn diagram illustrating the overlap of DEMs among three defined stress stages. These stages are: ER, Early Response State: (DEMs from T1vsCK); LA, Long-term Adaptation State: (DEMs from T4vsCK); RS, Recovered State: (DEMs from TRvsCK); SP, Stress Progression Process: (DEMs from T4vsT1); RP, Recovery Process: (DEMs from TRvsT1 and TRvsT4). **d** Heatmap of hierarchical clustering of DEMs specifically within the five stages defined in (**c**). Rows represent different treatment points and columns represent miRNAs at each stage (ER, LA, RS, SP, RP defined in c)

To investigate the dynamic miRNA response to salt stress, we identified DEMs across six pairwise comparisons (Fig. 2a, Additional file 1: Table S7). Compared to the control (CK), 6, 17, and 17 DEMs were identified in T1, T4, and TR, respectively. Among the stress-stage comparisons, T1vsT4, T1vsTR, and T4vsTR yielded 15, 11, and 7 DEMs, respectively, with a general preponderance of up-regulated miRNAs. Hierarchical clustering of all DEMs revealed four major expression trends, with a predominant pattern of up-regulation following salt stress (Fig. 2b, Additional file 1: Table S8).

To capture both the distinct physiological states and the dynamic transitions during salt stress response, we categorized these six pairwise comparisons into five biologically meaningful groups (Fig. 2c, Additional file 1: Table S9): Early Response State (ER): T1vsCK (6 DEMs); Long-term Adaptation State (LA): T4vsCK (17 DEMs); Recovered State (RS): TRvsCK (17 DEMs); Stress Progression Process (SP): T4vsT1 ( 15 DEMs); and Recovery Process (RP): union of TRvsT1 and TRvsT4 (18 DEMs after removing duplicates). This categorization revealed a marked increase in responsive miRNAs from ER (6) to LA (17), with substantial sets of miRNAs involved in SP (15) and RP (18). Notably, 17 miRNAs remained differentially expressed in the recovered state (RS). No miRNAs were shared across all five stages, indicating highly phase-specific regulatory programs.

Analysis of miRNA family distribution revealed distinct phase-specific patterns (Fig. 2d; Additional file 1: Table S9). miR6462 and miR6476 were unique to ER; miR477 was specific to RS; miR169 was specific to SP; and miR6457 was specific to RP. miR3627 was active in both ER and LA, while miR536 was active in both LA and RS. Notably, miR395 family members were distributed across all stages except ER, suggesting a sustained role in stress adaptation (Fig. 2d; Additional file 1: Table S9). Overall, most known miRNA families exhibited increased expression levels during long-term stress and recovery phases (LA, SP, RP, RS) compared to ER, whereas novel miRNAs showed more variable expression patterns.

### Transcriptome dynamics of *P. yunnanensis* reveals stage-specific responses to salt stress

We performed transcriptome sequencing on the same samples, generating 12 mRNA libraries with 78–84 million clean reads each (Additional file 1: Table S2). Principal component analysis (PCA) revealed clear separation among treatment points, with PC1 and PC2 explaining 28.25% and 17.21% of the total variance, respectively. The CK and T1 points clustered closely, while the T4 and TR points were distinctly separated, with TR being the most divergent (Additional file 2: Fig. S2).

**Fig. 3:**
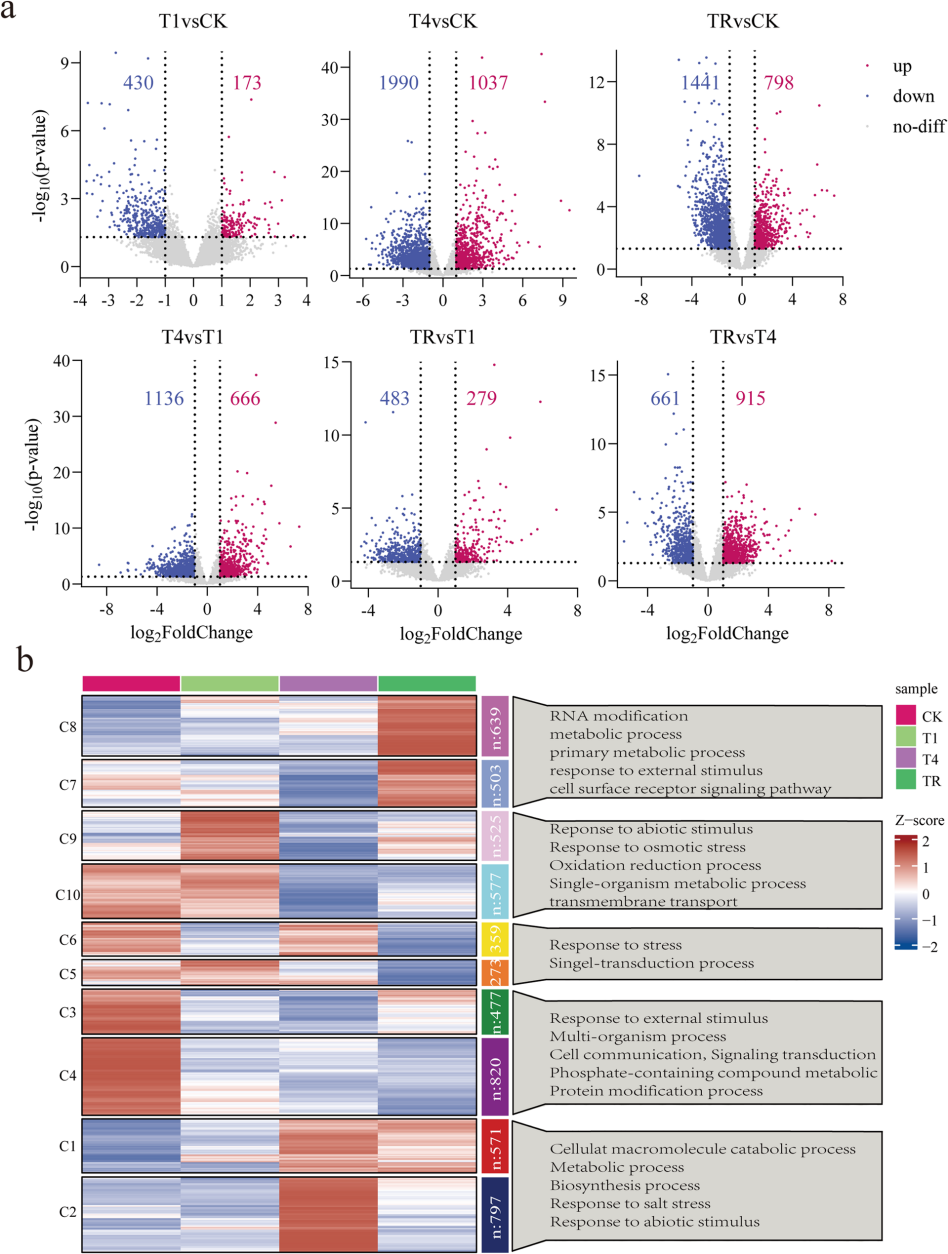
Identification and functional clustering of differentially expressed genes (DEGs) in *P. yunnanensis* under salt stress.** a** Volcano plots showing differentially expressed genes (DEGs) in six pairwise comparisons (T1vsCK, T4vsCK, TRvsCK, T4vsT1, TRvsT1, and TRvsT4). The x-axis represents log_2_FoldChange, and the y-axis represents -log_10_(*p*-value). Red dots indicate significantly up-regulated genes (|log₂FC| ≥ 1, *p*-value < 0.05), blue dots indicate significantly down-regulated genes (|log₂FC| ≥ 1, *p*-value < 0.05), and gray dots represent genes with no significant difference. **b** Left: Heatmap of hierarchical clustering of all DEGs across four treatment points (CK, T1, T4, TR). Genes were grouped into ten clusters (C1-C10) based on their expression patterns. The color scale represents Z-score normalized expression levels. Middle panel: Bar plots showing the number of DEGs in each cluster. Right panel: Significantly enriched Gene Ontology (GO) terms for each cluster, with representative functional categories listed

We identified 21,612 mRNAs across four points (Additional file 2: Fig. S1b). Differential expression analysis indicates that the scale of transcriptional reprogramming was dependent on stress duration. While only 603 DEGs were detected in T1vsCK, this number increased dramatically to 3,027 in T4vsCK and 2,239 DEGs in TRvsCK (Fig. 3b, Additional file 1: Table S10). Notably, the second group (T4vsCK) was characterized by a dominance of down-regulated genes (1,037 up vs. 1,990 down), a trend that persisted into the third group (TRvsCK: 798 up vs. 1441 down). Comparisons between stress stages (e.g., T4vsT1, TRvsT1) further confirmed this extensive transcriptome restructuring.

Hierarchical clustering of all DEGs delineated ten co-expression clusters with distinct temporal patterns (Fig. 3b, Additional file 1: Table S11). GO enrichment analysis revealed stage-specific functional themes: processes like signal transduction and protein modification were predominant in CK and T1; biosynthesis and response to salt stress were highly enriched in T4; while metabolic processes and RNA modification were characteristic of TR (Additional file 1: Table S12). Molecular functions were primarily enriched in ion binding, transcription factor activity, and ubiquitin-protein ligase activity, and cellular components were significantly associated with plastids and chloroplasts.

### KEGG enrichment highlights the dominance of metabolic and signaling pathways in stage-specific salt stress responses

**Fig. 4:**
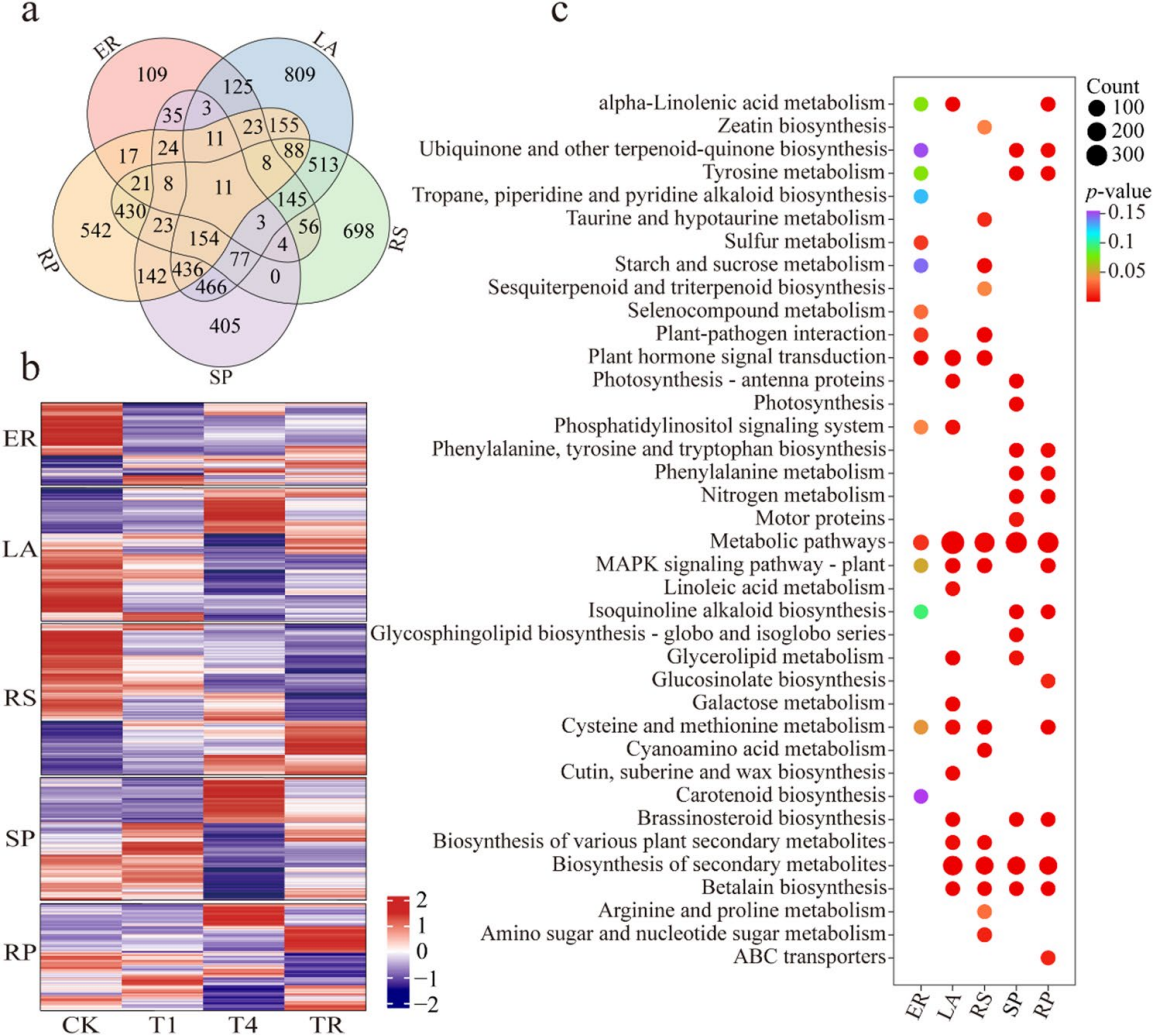
Stage-specific transcriptome dynamics and functional enrichment analysis of DEGs in *P. yunnanensis* under salt stress.** a** Venn diagram illustrating the overlap of DEGs among the five biologically defined phases: Early Response State (ER, T1vsCK), Long-term Adaptation State (LA, T4vsCK), Recovered State (RS, TRvsCK), Stress Progression Process (SP, T4vsT1), and Recovery Process (RP, union of TRvsT1 and TRvsT4). **b** Heatmap of hierarchical clustering of all DEGs across four treatment points (CK, T1, T4, TR), with genes grouped according to their expression patterns in the five phases defined in (**a**). Rows represent genes and columns represent treatment points. The color scale represents normalized expression levels, illustrating stage-specific expression trends. **c** Bubble plot of KEGG pathway enrichment analysis for DEGs in each of the five phases. The y-axis shows the pathway name, and the x-axis represent the five phases. Bubble size indicates the number of DEGs mapped to a given pathway, and bubble color represents the significance level (-log_10_(*p*-value))

To delineate the core transcriptional events, we consolidated DEGs into five biologically defined stages: Early Response State (ER, T1vsCK; 603 DEGs), Long-term Adaptation State (LA, T4vsCK; 3,027 DEGs), Recovered State (RS, TRvsCK; 2,239 DEGs), Stress Progression Process (SP, T4vsT1; 1,802 DEGs), and Recovery Process (RP, union of TRvsT1 and TRvsT4; 2,093 DEGs) (Fig. 4a, Additional file 1: Table S13).

Venn diagram analysis revealed the overlapping and unique DEGs across these five phases (Fig. 4a). The ER contained 603 DEGs, of which 109 genes (18.1%) were phase-specific. ER shared 329 genes with Long-term Adaptation State (LA), 196 genes with Recovered State (RS), 99 genes with Stress Progression Process (SP), and 123 genes with Recovery Process (RP). LA exhibited the largest number of DEGs (3,027), including 809 phase-specific genes (26.7%). LA shared substantial numbers with SP (1,161 genes), RP (886 genes), RS (999 genes), and ER (329 genes). SP comprised 1,802 DEGs, with 405 phase-specific genes (22.5%). Notably, SP shared 1,161 genes with LA and 809 genes with RP, as well as 280 genes with RS and 99 genes with ER, reflecting its transitional nature between stress establishment and recovery. RP contained 2,093 DEGs, including 542 phase-specific genes (25.9%). RP shared 809 genes with SP, 886 genes with LA, 655 genes with RS, and 123 genes with ER, suggesting both active recovery mechanisms and persistent stress signatures. RS retained 2,239 DEGs compared to the initial control, with 698 phase-specific genes (31.2%). RS shared 999 genes with LA, 655 genes with RP, 280 genes with SP, and 196 genes with ER, indicating potential stress memory effects. Interestingly, only 11 genes were commonly expressed across all five phases, highlighting the highly stage-specific nature of the transcriptional reprogramming during salt stress adaptation (Fig. 4b).

KEGG pathway enrichment analysis revealed both common and stage-specific regulatory themes across the five phases (Fig. 4c, Additional file 1: Table S14). Metabolic pathways were consistently enriched in all phases, serving as a basal transcriptional program underlying salt stress adaptation. In the Early Response State (ER), pathways related to initial stress perception and signal transduction, including plant hormone signal transduction and MAPK signaling pathway - plant, showed significant enrichment (*p*-value < 0.05), reflecting rapid activation of stress signaling cascades upon salt exposure. The Stress Progression Process (SP) exhibited a broad activation of metabolic pathways, with significant enrichment of glycerolipid metabolism, and biosynthesis of various secondary metabolites, indicating active metabolic remodeling during the transition from short-term to long-term stress. The Long-term Adaptation State (LA) was characterized by prominent enrichment of primary metabolism pathways, particularly cysteine and methionine metabolism, alongside sustained enrichment of plant hormone signal transduction and MAPK signaling pathway. In the Recovery Process (RP), pathways associated with membrane remodeling and defense compound production, such as alpha-Linolenic acid metabolism and phenylpropanoid biosynthesis, were significantly enriched. The Recovered State (RS) retained enrichment of multiple metabolic pathways, including cysteine and methionine metabolism, biosynthesis of various secondary metabolites, arginine and proline metabolism, and amino sugar and nucleotide sugar metabolism, alongside persistent plant hormone signal transduction. Dynamic regulation was observed for photosynthesis-related pathways: photosynthesis and photosynthesis-antenna proteins were enriched specifically in LA and SP, indicating long-term adjustments of photosynthetic capacity during stress adaptation, with recovery of these pathways after stress relief.

### Integrated analysis of miRNA-mRNA interactions reveals core regulatory circuits in salt stress response

**Fig. 5:**
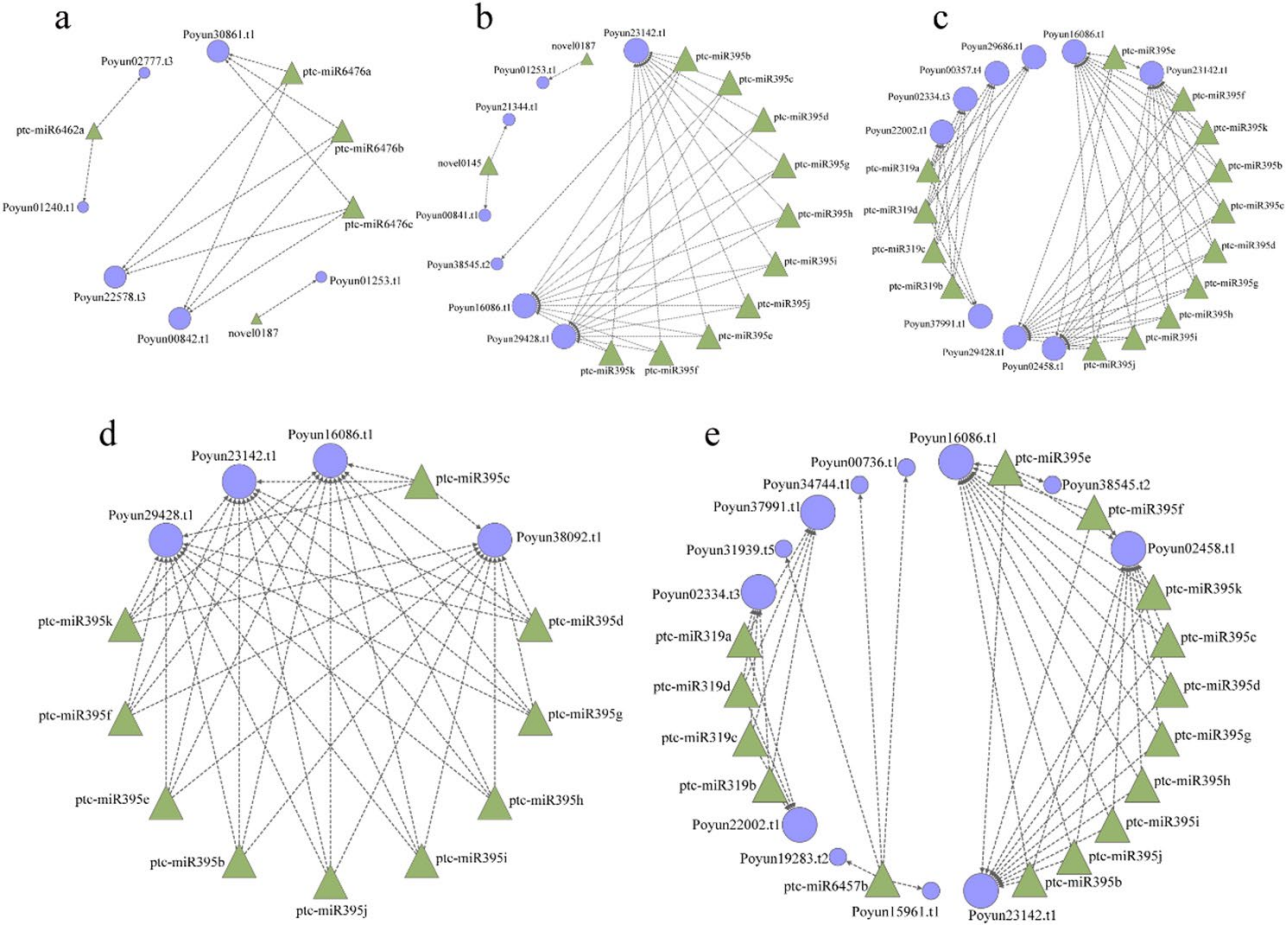
Stage-specific miRNA-mRNA regulatory networks in *P. yunnanensis* under salt stress. Networks were constructed for five biologically defined phases: **a** Early Response (ER, T1vsCK), **b** Long-term Adaptation (LA, T4vsCK), **c** Recovered State (RS, TRvsCK), **d** Stress Progression (SP, T4vsT1), and **e** Recovery Process (RP, union of TRvsT1 and TRvsT4). Green trangles represent miRNAs and blue circles represent target mRNAs. Edges represent high-confidence miRNA-mRNA interactions identified by both psRobot and TargetFinder, with anti-correlated expression patterns (Pearson correlation coefficient < -0.8, *p*-value < 0.05)

To decipher the post-transcriptional regulatory network, we integrated miRNA and mRNA expression data to identify significant miRNA-mRNA interaction pairs across the five stress stages (Additional file 1: Table S15, Table S16). The complexity of the network increased with stress and recovery, with 12 pairs (involving 5 miRNAs and 6 targets) in ER, 34 pairs (12 miRNAs, 7 targets) in LA, 59 pairs (14 miRNAs, 9 targets) in RS, 40 pairs (10 miRNAs, 4 targets) in SP and 48 pairs (15 miRNAs, 12 targets) in RP (Fig. 5; Table 1).

**Table 1:** Key miRNA-mRNA interaction in *P. yunnanensis* under salt stress

Group	miRNA	regulate-miRNA	Target-mRNA	regulate-mRNA	description	perr
ER	ptc-miR6476a	up	*Poyun30861.t1*	down	-	-1.00
ER	ptc-miR6476b	up	*Poyun30861.t1*	down	-	-1.00
ER	ptc-miR6476c	up	*Poyun30861.t1*	down	-	-1.00
ER	novel0187	up	*Poyun01253.t1*	down	Leucine-rich repeat receptor-like protein kinase	-1.00
ER	ptc-miR6476a	up	*Poyun00842.t1*	down	Domain of unknown function (DUF4228)	-1.00
ER	ptc-miR6476a	up	*Poyun22578.t3*	down	The GINS complex plays an essential role in the initiation of DNA replication	-1.00
ER	ptc-miR6476b	up	*Poyun00842.t1*	down	Domain of unknown function (DUF4228)	-1.00
ER	ptc-miR6476b	up	*Poyun22578.t3*	down	The GINS complex plays an essential role in the initiation of DNA replication	-1.00
ER	ptc-miR6476c	up	*Poyun00842.t1*	down	Domain of unknown function (DUF4228)	-1.00
ER	ptc-miR6476c	up	*Poyun22578.t3*	down	The GINS complex plays an essential role in the initiation of DNA replication	-1.00
ER	ptc-miR6462a	down	*Poyun01240.t1*	down	NO-associated protein 1, chloroplastic	-1.00
ER	ptc-miR6462a	down	*Poyun02777.t3*	down	Belongs to the HisA HisF family	-1.00
LA	ptc-miR395b	up	*Poyun23142.t1*	down	ATP sulfurylase 1	-1.00
LA	ptc-miR395c	up	*Poyun23142.t1*	down	ATP sulfurylase 1	-1.00
LA	ptc-miR395d	up	*Poyun23142.t1*	down	ATP sulfurylase 1	-1.00
LA	ptc-miR395g	up	*Poyun23142.t1*	down	ATP sulfurylase 1	-1.00
LA	ptc-miR395h	up	*Poyun23142.t1*	down	ATP sulfurylase 1	-1.00
LA	ptc-miR395i	up	*Poyun23142.t1*	down	ATP sulfurylase 1	-1.00
LA	ptc-miR395j	up	*Poyun23142.t1*	down	ATP sulfurylase 1	-1.00
LA	ptc-miR395e	up	*Poyun23142.t1*	down	ATP sulfurylase 1	-1.00
LA	ptc-miR395f	up	*Poyun23142.t1*	down	ATP sulfurylase 1	-1.00
LA	ptc-miR395k	up	*Poyun23142.t1*	down	ATP sulfurylase 1	-1.00
LA	ptc-miR395b	up	*Poyun29428.t1*	down	Sulfate transporter	-1.00
LA	ptc-miR395c	up	*Poyun29428.t1*	down	Sulfate transporter	-1.00
LA	ptc-miR395d	up	*Poyun29428.t1*	down	Sulfate transporter	-1.00
LA	ptc-miR395g	up	*Poyun29428.t1*	down	Sulfate transporter	-1.00
LA	ptc-miR395h	up	*Poyun29428.t1*	down	Sulfate transporter	-1.00
LA	ptc-miR395i	up	*Poyun29428.t1*	down	Sulfate transporter	-1.00
LA	ptc-miR395j	up	*Poyun29428.t1*	down	Sulfate transporter	-1.00
LA	ptc-miR395e	up	*Poyun29428.t1*	down	Sulfate transporter	-1.00
LA	ptc-miR395f	up	*Poyun29428.t1*	down	Sulfate transporter	-1.00
LA	ptc-miR395k	up	*Poyun29428.t1*	down	Sulfate transporter	-1.00
LA	ptc-miR395b	up	*Poyun16086.t1*	down	ATP sulfurylase 1	-1.00
LA	ptc-miR395b	up	*Poyun38545.t2*	down	Belongs to the ubiquitin-activating E1 family	-1.00
LA	ptc-miR395c	up	*Poyun16086.t1*	down	ATP sulfurylase 1	-1.00
LA	ptc-miR395d	up	*Poyun16086.t1*	down	ATP sulfurylase 1	-1.00
LA	ptc-miR395g	up	*Poyun16086.t1*	down	ATP sulfurylase 1	-1.00
LA	ptc-miR395h	up	*Poyun16086.t1*	down	ATP sulfurylase 1	-1.00
LA	ptc-miR395i	up	*Poyun16086.t1*	down	ATP sulfurylase 1	-1.00
LA	ptc-miR395j	up	*Poyun16086.t1*	down	ATP sulfurylase 1	-1.00
LA	ptc-miR395e	up	*Poyun16086.t1*	down	ATP sulfurylase 1	-1.00
LA	ptc-miR395f	up	*Poyun16086.t1*	down	ATP sulfurylase 1	-1.00
LA	ptc-miR395k	up	*Poyun16086.t1*	down	ATP sulfurylase 1	-1.00
LA	novel0145	up	*Poyun00841.t1*	down	Glutaredoxin	-1.00
LA	novel0145	up	*Poyun21344.t1*	down	B-cell receptor-associated protein	-1.00
LA	novel0187	up	*Poyun01253.t1*	down	Leucine-rich repeat receptor-like protein kinase	-1.00
RS	ptc-miR395e	up	*Poyun16086.t1*	down	ATP sulfurylase 1	-1.00
RS	ptc-miR395e	up	*Poyun23142.t1*	down	ATP sulfurylase 1	-1.00
RS	ptc-miR395f	up	*Poyun16086.t1*	down	ATP sulfurylase 1	-1.00
RS	ptc-miR395f	up	*Poyun23142.t1*	down	ATP sulfurylase 1	-1.00
RS	ptc-miR395k	up	*Poyun16086.t1*	down	ATP sulfurylase 1	-1.00
RS	ptc-miR395k	up	*Poyun23142.t1*	down	ATP sulfurylase 1	-1.00
RS	ptc-miR395b	up	*Poyun16086.t1*	down	ATP sulfurylase 1	-1.00
RS	ptc-miR395b	up	*Poyun23142.t1*	down	ATP sulfurylase 1	-1.00
RS	ptc-miR395c	up	*Poyun16086.t1*	down	ATP sulfurylase 1	-1.00
RS	ptc-miR395c	up	*Poyun23142.t1*	down	ATP sulfurylase 1	-1.00
RS	ptc-miR395d	up	*Poyun16086.t1*	down	ATP sulfurylase 1	-1.00
RS	ptc-miR395d	up	*Poyun23142.t1*	down	ATP sulfurylase 1	-1.00
RS	ptc-miR395g	up	*Poyun16086.t1*	down	ATP sulfurylase 1	-1.00
RS	ptc-miR395g	up	*Poyun23142.t1*	down	ATP sulfurylase 1	-1.00
RS	ptc-miR395h	up	*Poyun16086.t1*	down	ATP sulfurylase 1	-1.00
RS	ptc-miR395h	up	*Poyun23142.t1*	down	ATP sulfurylase 1	-1.00
RS	ptc-miR395i	up	*Poyun16086.t1*	down	ATP sulfurylase 1	-1.00
RS	ptc-miR395i	up	*Poyun23142.t1*	down	ATP sulfurylase 1	-1.00
RS	ptc-miR395j	up	*Poyun16086.t1*	down	ATP sulfurylase 1	-1.00
RS	ptc-miR395j	up	*Poyun23142.t1*	down	ATP sulfurylase 1	-1.00
RS	ptc-miR395e	up	*Poyun02458.t1*	down	Fusaric acid resistance protein-like	-1.00
RS	ptc-miR395e	up	*Poyun29428.t1*	down	Sulfate transporter	-1.00
RS	ptc-miR395f	up	*Poyun02458.t1*	down	Fusaric acid resistance protein-like	-1.00
RS	ptc-miR395f	up	*Poyun29428.t1*	down	Sulfate transporter	-1.00
RS	ptc-miR395k	up	*Poyun02458.t1*	down	Fusaric acid resistance protein-like	-1.00
RS	ptc-miR395k	up	*Poyun29428.t1*	down	Sulfate transporter	-1.00
RS	ptc-miR395b	up	*Poyun02458.t1*	down	Fusaric acid resistance protein-like	-1.00
RS	ptc-miR395b	up	*Poyun29428.t1*	down	Sulfate transporter	-1.00
RS	ptc-miR395c	up	*Poyun02458.t1*	down	Fusaric acid resistance protein-like	-1.00
RS	ptc-miR395c	up	*Poyun29428.t1*	down	Sulfate transporter	-1.00
RS	ptc-miR395d	up	*Poyun02458.t1*	down	Fusaric acid resistance protein-like	-1.00
RS	ptc-miR395d	up	*Poyun29428.t1*	down	Sulfate transporter	-1.00
RS	ptc-miR395g	up	*Poyun02458.t1*	down	Fusaric acid resistance protein-like	-1.00
RS	ptc-miR395g	up	*Poyun29428.t1*	down	Sulfate transporter	-1.00
RS	ptc-miR395h	up	*Poyun02458.t1*	down	Fusaric acid resistance protein-like	-1.00
RS	ptc-miR395h	up	*Poyun29428.t1*	down	Sulfate transporter	-1.00
RS	ptc-miR395i	up	*Poyun02458.t1*	down	Fusaric acid resistance protein-like	-1.00
RS	ptc-miR395i	up	*Poyun29428.t1*	down	Sulfate transporter	-1.00
RS	ptc-miR395j	up	*Poyun02458.t1*	down	Fusaric acid resistance protein-like	-1.00
RS	ptc-miR395j	up	*Poyun29428.t1*	down	Sulfate transporter	-1.00
RS	ptc-miR319b	down	*Poyun37991.t1*	up	Transcription factor	-1.00
RS	ptc-miR319c	down	*Poyun37991.t1*	up	Transcription factor	-1.00
RS	ptc-miR319d	down	*Poyun37991.t1*	up	Transcription factor	-1.00
RS	ptc-miR319a	down	*Poyun37991.t1*	up	Transcription factor	-1.00
RS	ptc-miR319b	down	*Poyun22002.t1*	up	-	-1.00
RS	ptc-miR319c	down	*Poyun22002.t1*	up	-	-1.00
RS	ptc-miR319d	down	*Poyun22002.t1*	up	-	-1.00
RS	ptc-miR319a	down	*Poyun22002.t1*	up	-	-1.00
RS	ptc-miR319b	down	*Poyun02334.t3*	up	Transcription factor	-1.00
RS	ptc-miR319c	down	*Poyun02334.t3*	up	Transcription factor	-1.00
RS	ptc-miR319d	down	*Poyun02334.t3*	up	Transcription factor	-1.00
RS	ptc-miR319a	down	*Poyun02334.t3*	up	Transcription factor	-1.00
RS	ptc-miR319b	down	*Poyun00357.t4*	up	transcription factor	-1.00
RS	ptc-miR319c	down	*Poyun00357.t4*	up	transcription factor	-1.00
RS	ptc-miR319d	down	*Poyun00357.t4*	up	transcription factor	-1.00
RS	ptc-miR319a	down	*Poyun00357.t4*	up	transcription factor	-1.00
RS	ptc-miR319c	down	*Poyun29686.t1*	up	N/A	-1.00
RS	ptc-miR319d	down	*Poyun29686.t1*	up	N/A	-1.00
RS	ptc-miR319a	down	*Poyun29686.t1*	up	N/A	-1.00
SP	ptc-miR395c	up	*Poyun16086.t1*	down	ATP sulfurylase 1	-1.00
SP	ptc-miR395c	up	*Poyun38092.t1*	down	Fusaric acid resistance protein-like	-1.00
SP	ptc-miR395d	up	*Poyun38092.t1*	down	Fusaric acid resistance protein-like	-1.00
SP	ptc-miR395g	up	*Poyun38092.t1*	down	Fusaric acid resistance protein-like	-1.00
SP	ptc-miR395h	up	*Poyun38092.t1*	down	Fusaric acid resistance protein-like	-1.00
SP	ptc-miR395i	up	*Poyun38092.t1*	down	Fusaric acid resistance protein-like	-1.00
SP	ptc-miR395j	up	*Poyun38092.t1*	down	Fusaric acid resistance protein-like	-1.00
SP	ptc-miR395b	up	*Poyun38092.t1*	down	Fusaric acid resistance protein-like	-1.00
SP	ptc-miR395e	up	*Poyun38092.t1*	down	Fusaric acid resistance protein-like	-1.00
SP	ptc-miR395f	up	*Poyun38092.t1*	down	Fusaric acid resistance protein-like	-1.00
SP	ptc-miR395k	up	*Poyun38092.t1*	down	Fusaric acid resistance protein-like	-1.00
SP	ptc-miR395c	up	*Poyun29428.t1*	down	Sulfate transporter	-1.00
SP	ptc-miR395d	up	*Poyun29428.t1*	down	Sulfate transporter	-1.00
SP	ptc-miR395g	up	*Poyun29428.t1*	down	Sulfate transporter	-1.00
SP	ptc-miR395h	up	*Poyun29428.t1*	down	Sulfate transporter	-1.00
SP	ptc-miR395i	up	*Poyun29428.t1*	down	Sulfate transporter	-1.00
SP	ptc-miR395j	up	*Poyun29428.t1*	down	Sulfate transporter	-1.00
SP	ptc-miR395b	up	*Poyun29428.t1*	down	Sulfate transporter	-1.00
SP	ptc-miR395e	up	*Poyun29428.t1*	down	Sulfate transporter	-1.00
SP	ptc-miR395f	up	*Poyun29428.t1*	down	Sulfate transporter	-1.00
SP	ptc-miR395k	up	*Poyun29428.t1*	down	Sulfate transporter	-1.00
SP	ptc-miR395c	up	*Poyun23142.t1*	down	ATP sulfurylase 1	-1.00
SP	ptc-miR395d	up	*Poyun23142.t1*	down	ATP sulfurylase 1	-1.00
SP	ptc-miR395g	up	*Poyun23142.t1*	down	ATP sulfurylase 1	-1.00
SP	ptc-miR395h	up	*Poyun23142.t1*	down	ATP sulfurylase 1	-1.00
SP	ptc-miR395i	up	*Poyun23142.t1*	down	ATP sulfurylase 1	-1.00
SP	ptc-miR395j	up	*Poyun23142.t1*	down	ATP sulfurylase 1	-1.00
SP	ptc-miR395b	up	*Poyun23142.t1*	down	ATP sulfurylase 1	-1.00
SP	ptc-miR395e	up	*Poyun23142.t1*	down	ATP sulfurylase 1	-1.00
SP	ptc-miR395f	up	*Poyun23142.t1*	down	ATP sulfurylase 1	-1.00
SP	ptc-miR395k	up	*Poyun23142.t1*	down	ATP sulfurylase 1	-1.00
SP	ptc-miR395d	up	*Poyun16086.t1*	down	ATP sulfurylase 1	-1.00
SP	ptc-miR395g	up	*Poyun16086.t1*	down	ATP sulfurylase 1	-1.00
SP	ptc-miR395h	up	*Poyun16086.t1*	down	ATP sulfurylase 1	-1.00
SP	ptc-miR395i	up	*Poyun16086.t1*	down	ATP sulfurylase 1	-1.00
SP	ptc-miR395j	up	*Poyun16086.t1*	down	ATP sulfurylase 1	-1.00
SP	ptc-miR395b	up	*Poyun16086.t1*	down	ATP sulfurylase 1	-1.00
SP	ptc-miR395e	up	*Poyun16086.t1*	down	ATP sulfurylase 1	-1.00
SP	ptc-miR395f	up	*Poyun16086.t1*	down	ATP sulfurylase 1	-1.00
SP	ptc-miR395k	up	*Poyun16086.t1*	down	ATP sulfurylase 1	-1.00
RP	ptc-miR395e	up	*Poyun16086.t1*	down	ATP sulfurylase 1	-1.00
RP	ptc-miR395e	up	*Poyun38545.t2*	down	Belongs to the ubiquitin-activating E1 family	-1.00
RP	ptc-miR395f	up	*Poyun16086.t1*	down	ATP sulfurylase 1	-1.00
RP	ptc-miR395e	up	*Poyun02458.t1*	down	Fusaric acid resistance protein-like	-1.00
RP	ptc-miR395f	up	*Poyun02458.t1*	down	Fusaric acid resistance protein-like	-1.00
RP	ptc-miR395k	up	*Poyun02458.t1*	down	Fusaric acid resistance protein-like	-1.00
RP	ptc-miR395c	up	*Poyun02458.t1*	down	Fusaric acid resistance protein-like	-1.00
RP	ptc-miR395d	up	*Poyun02458.t1*	down	Fusaric acid resistance protein-like	-1.00
RP	ptc-miR395g	up	*Poyun02458.t1*	down	Fusaric acid resistance protein-like	-1.00
RP	ptc-miR395h	up	*Poyun02458.t1*	down	Fusaric acid resistance protein-like	-1.00
RP	ptc-miR395i	up	*Poyun02458.t1*	down	Fusaric acid resistance protein-like	-1.00
RP	ptc-miR395j	up	*Poyun02458.t1*	down	Fusaric acid resistance protein-like	-1.00
RP	ptc-miR395b	up	*Poyun02458.t1*	down	Fusaric acid resistance protein-like	-1.00
RP	ptc-miR395k	up	*Poyun16086.t1*	down	ATP sulfurylase 1	-1.00
RP	ptc-miR395c	up	*Poyun16086.t1*	down	ATP sulfurylase 1	-1.00
RP	ptc-miR395d	up	*Poyun16086.t1*	down	ATP sulfurylase 1	-1.00
RP	ptc-miR395g	up	*Poyun16086.t1*	down	ATP sulfurylase 1	-1.00
RP	ptc-miR395h	up	*Poyun16086.t1*	down	ATP sulfurylase 1	-1.00
RP	ptc-miR395i	up	*Poyun16086.t1*	down	ATP sulfurylase 1	-1.00
RP	ptc-miR395j	up	*Poyun16086.t1*	down	ATP sulfurylase 1	-1.00
RP	ptc-miR395b	up	*Poyun16086.t1*	down	ATP sulfurylase 1	-1.00
RP	ptc-miR395e	up	*Poyun23142.t1*	down	ATP sulfurylase 1	-1.00
RP	ptc-miR395f	up	*Poyun23142.t1*	down	ATP sulfurylase 1	-1.00
RP	ptc-miR395k	up	*Poyun23142.t1*	down	ATP sulfurylase 1	-1.00
RP	ptc-miR395c	up	*Poyun23142.t1*	down	ATP sulfurylase 1	-1.00
RP	ptc-miR395d	up	*Poyun23142.t1*	down	ATP sulfurylase 1	-1.00
RP	ptc-miR395g	up	*Poyun23142.t1*	down	ATP sulfurylase 1	-1.00
RP	ptc-miR395h	up	*Poyun23142.t1*	down	ATP sulfurylase 1	-1.00
RP	ptc-miR395i	up	*Poyun23142.t1*	down	ATP sulfurylase 1	-1.00
RP	ptc-miR395j	up	*Poyun23142.t1*	down	ATP sulfurylase 1	-1.00
RP	ptc-miR395b	up	*Poyun23142.t1*	down	ATP sulfurylase 1	-1.00
RP	ptc-miR6457b	down	*Poyun15961.t1*	up	MazG nucleotide pyrophosphohydrolase domain	-1.00
RP	ptc-miR6457b	down	*Poyun19283.t2*	up	-	-1.00
RP	ptc-miR319b	down	*Poyun22002.t1*	up	-	-1.00
RP	ptc-miR319c	down	*Poyun22002.t1*	up	-	-1.00
RP	ptc-miR319d	down	*Poyun22002.t1*	up	-	-1.00
RP	ptc-miR319a	down	*Poyun22002.t1*	up	-	-1.00
RP	ptc-miR319b	down	*Poyun02334.t3*	up	Transcription factor	-1.00
RP	ptc-miR319c	down	*Poyun02334.t3*	up	Transcription factor	-1.00
RP	ptc-miR319d	down	*Poyun02334.t3*	up	Transcription factor	-1.00
RP	ptc-miR319a	down	*Poyun02334.t3*	up	Transcription factor	-1.00
RP	ptc-miR6457b	down	*Poyun31939.t5*	up	cysteine protease	-1.00
RP	ptc-miR319b	down	*Poyun37991.t1*	up	Transcription factor	-1.00
RP	ptc-miR319c	down	*Poyun37991.t1*	up	Transcription factor	-1.00
RP	ptc-miR319d	down	*Poyun37991.t1*	up	Transcription factor	-1.00
RP	ptc-miR319a	down	*Poyun37991.t1*	up	Transcription factor	-1.00
RP	ptc-miR6457b	down	*Poyun34744.t1*	up	Belongs to the protein kinase superfamily. Ser Thr protein kinase family	-1.00
RP	ptc-miR6457b	down	*Poyun00736.t1*	up	N/A	-1.00

Group: five biologically defined stages

perr: Pearson correlation coefficient between the expression values of miRNA and mRNA during the testing stage

*ER* Early Response State, *LA* Long-term Adaptation State, *RS* Recovered State, *SP* Stress Progression Process, *RP* Recovery Process

The regulatory networks exhibited distinct functional compositions depending on the stress stage (Additional file 1: Table S15, Fig. 5). In the Early Response State (ER), the miRNA-targeted genes were predominantly enriched in signal perception and transduction, including leucine-rich repeat (LRR) domain-containing proteins and protein kinases, as well as components involved in DNA replication protection, such as the SLD5 subunit of the GINS complex. In the Long-term Adaptation State (LA), the network expanded to include genes associated with sulfate assimilation and redox homeostasis, such as ATP sulfurylase, sulfate transporters, and glutaredoxins. The Stress Progression Process (SP) showed a more focused regulatory module centered on sulfate metabolism. During the Recovery Process (RP), the network became more complex, encompassing protein degradation pathways (ubiquitin-activating enzyme E1), nucleotide homeostasis regulators (MazG), transcription factors (MYB), and signaling proteins (LRR-containing proteins). In the Recovered State (RS), the network retained elements of sulfate metabolism and transcriptional regulation (MYB), indicating persistent metabolic adjustments that may contribute to stress memory.

**Fig. 6:**
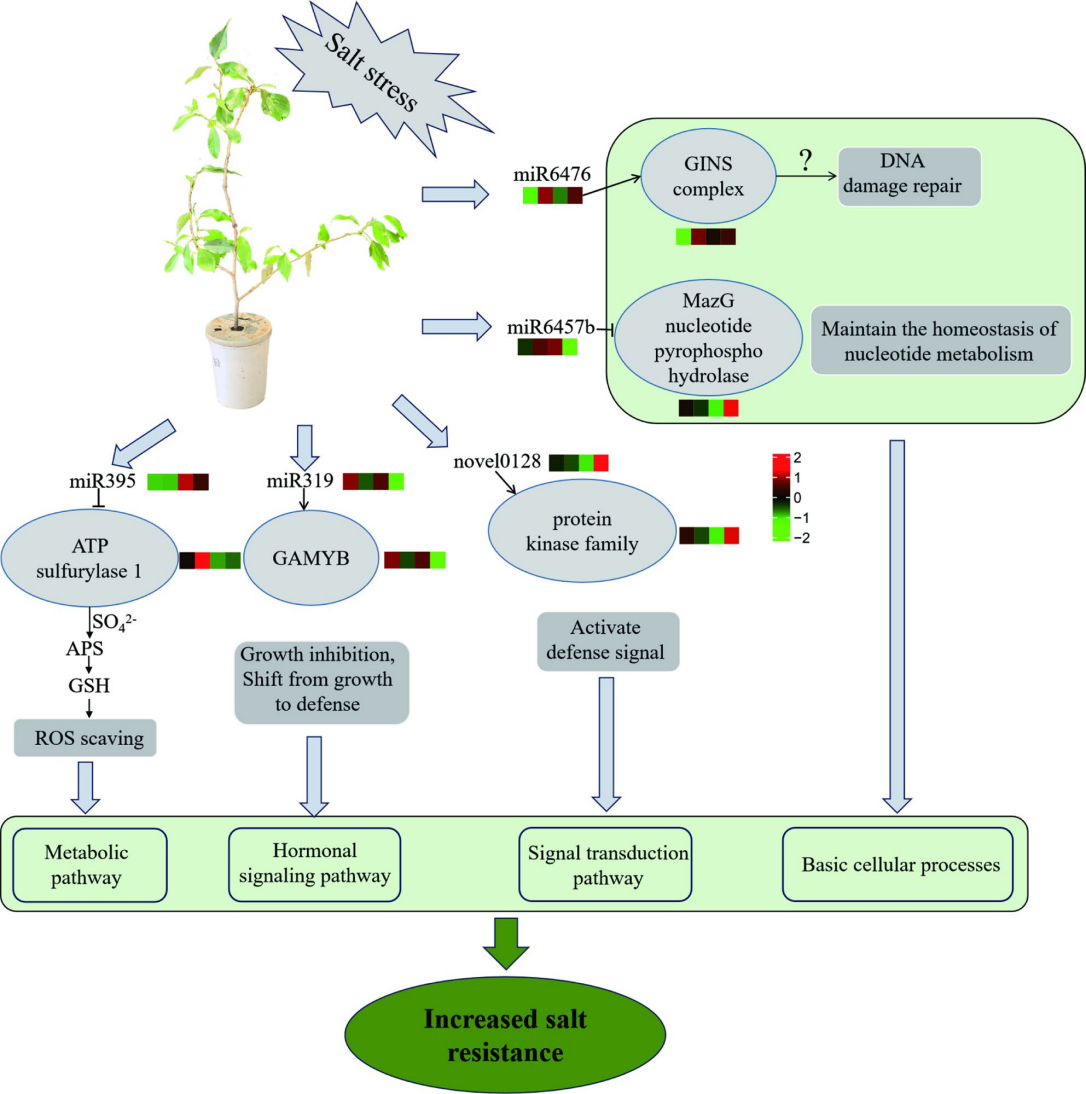
A proposed model for miRNA-mediated regulatory networks in the salt stress response of *P. yunnanensis*. The model integrates key miRNA-mRNA regulatory pairs identified in this study. Colored bars represent the expression trends of miRNAs and their target genes across the four treatment points (CK, T1, T4, TR). Arrows denote positive regulation, and T-bars denote negative regulation. Target genes (blue ellipses) and their putative roles in stress adaptation (blue boxes) are shown. This network highlights miRNA regulation in processes such as antioxidant synthesis (via ptc-miR395-APS1), growth-defense balance (via ptc-miR319-MYB), and DNA integrity maintenance (via ptc-miR6476-GINS)

Based on these core regulatory circuits, we propose a comprehensive model illustrating how miRNA-mediated post-transcriptional regulation coordinates key biological processes during salt stress adaptation in *P. yunnanensis* (Fig. 6). Central to this model is the ptc-miR395-APS1 circuit, which modulates sulfate assimilation and antioxidant synthesis during long-term stress and recovery. The ptc-miR319-MYB module acts as a putative growth-defense switch, with down-regulation of both miRNA and its target suggesting coordinated inhibition of growth to prioritize stress resources. Additionally, novel regulatory circuits were identified: novel0128 targeting a serine/threonine protein kinase, potentially modulating signal transduction; ptc-miR6457b-MazG involved in nucleotide pool homeostasis; and ptc-miR6476-GINS implicated in protecting DNA replication fidelity during early salt response (Additional file 1: Table S15). This model highlights the multifaceted roles of miRNAs in coordinating antioxidant defense, signaling, growth regulation, and genome stability under salt stress.

### Validation of sequencing data by qRT-PCR

The expression patterns of three randomly selected three miRNA-mRNA pairs from the sequencing data were validated by qRT-PCR. The qRT-PCR results showed high consistency with the high-throughput sequencing data (Additional file 2: Fig. S3), confirming the reliability of our transcriptome profiles.

## Discussion

Salt stress represents a major constraint on global forestry productivity and ecosystem stability, posing a significant threat to plant growth and metabolic homeostasis due to increasingly widespread soil salinization [26]. To survive and maintain growth under high salinity, plants have evolved sophisticated gene regulatory networks to orchestrate ion balance, osmotic adjustment, and photosynthetic protection [2]. Among these regulators, miRNA has emerged as key post-transcriptional modulator in response to various abiotic stresses, including salinity [13], heat [27], drought [28], and cold [29]. Although numerous salt-responsive miRNAs have been identified in herbaceous models [30, 31], genome-wide investigations in perennial woody plants, particularly *P. yunnanensis*, remain limited. Our study provides a comprehensive analysis of the miRNA and mRNA regulatory dynamics across five biologically defined phases of salt stress response in *P. yunnanensis*, unveiling novel insights into the temporal molecular mechanisms underlying salt adaptation in trees.

The length distribution of sRNAs in our libraries, with predominant 21 nt and 24 nt species (Fig. 1a), is consistent with patterns observed in other plant species such as *Halostachys caspica* [32], tomato [33], and *Brassica napus* [34]. The prevalence of 24 nt RNAs in unique reads reflects their associates with RNA-directed DNA methylation and silencing of repetitive elements [35, 36], while the abundance of 21 nt RNAs in total reads represents canonical miRNAs with potent regulatory functions [37]. The substantial proportion of unannotated sRNAs (Fig. 1b), echoes findings in other species and underscores the vast, yet unexplored, diversity of small RNAs in plants [32, 38, 39]. Our identification of 339 known miRNAs and 232 novel miRNAs substantially expands the miRNA repertoire of *P. yunnanensis*, providing a valuable resource for understanding post-transcriptional regulation in this ecologically important tree species.

The time-series analysis revealed that miRNA expression is highly dynamic and phase-specific in response to salt stress. The number of expressed miRNAs increased from control to long-term salt stress, and decreased during recovery, indicating that miRNA-mediated regulation is actively modulated throughout the stress response trajectory. The categorization of DEMs into five distinct phases—Early Response (ER), Long-term Adaptation (LA), Stress Progression (SP), Recovery Process (RP), and Recovered State (RS)—allowed us to dissect the temporal complexity of miRNA regulation. The limited overlap of DEMs between phases, with no miRNA common to all five phases, highlights a high degree of functional specialization, where different miRNA cohorts orchestrate distinct physiological transitions.

Several miRNA families exhibited striking phase-specificity: miR6462 and miR6476 were unique to ER, suggesting roles in initial stress perception and DNA protection; miR169 and miR6457 were specific to SP and RP, respectively, implicating them in stress progression and active recovery; while miR477 was unique to RS, potentially contributing to stress memory. The miR395 family, distributed across all stages except ER, appears to play a sustained role in stress adaptation, consistent with its conserved function in sulfate metabolism and redox homeostasis [16, 40]. The differential expression patterns of known versus novel miRNAs—with most known families showing increased expression during long-term stress and recovery phases, while novel miRNAs exhibited more variable patterns—suggests that conserved miRNA modules mediate core stress responses, whereas species-specific novel miRNAs may fine-tune adaptive strategies unique to *P. yunnanensis*.

The global transcriptomic response to salt stress in *P. yunnanensis* was profound and stage-specific. Principal component analysis (PCA) confirmed clear distinctions between four treatment points, with PCA revealing distinct separation among treatment points and TR showing the most divergent transcriptional state (Additional file 2: Fig. S2) This divergence suggests that recovery from salt stress does not merely represent a return to pre-stress conditions but involves lasting molecular adjustments. GO enrichment analysis revealed a logical shift in biological priorities across phases: from early signal transduction in CK and T1, to active biosynthesis and stress response in T4, and culminating in metabolic reconfiguration and RNA modification during TR (Additional file 1: Table S12). This layered transcriptional strategy underscores a sophisticated adaptation from rapid signaling to sustained physiological remodeling [17].

KEGG pathway enrichment analysis across the five phases revealed both common and stage-specific regulatory themes (Fig. 4c). Metabolic pathways were consistently enriched in all phases, serving as a basal transcriptional program. In ER, pathways related to initial stress perception—plant hormone signal transduction and MAPK signaling pathway—were significantly enriched, reflecting rapid activation of stress signaling cascades [41]. SP exhibited broad activation of metabolic pathways, including glycerolipid metabolism, and biosynthesis of various secondary metabolites [42], indicating active metabolic remodeling during the transition from short-term to long-term stress. LA was characterized by prominent enrichment of primary metabolism pathways, particularly cysteine and methionine metabolism and biosynthesis of amino acids, alongside sustained enrichment of hormone and MAPK signaling [43], suggesting that once stress adaptation is established, plants maintain both metabolic adjustments and signaling to sustain tolerance. In RP, pathways associated with membrane remodeling and defense compound production—alpha-Linolenic acid metabolism and phenylpropanoid biosynthesis [44]—were significantly enriched, pointing to active recovery mechanisms that repair stress-induced damage and reconstitute cellular structures. RS retained enrichment of multiple metabolic pathways, including cysteine and methionine metabolism, biosynthesis of various secondary metabolites, arginine and proline metabolism, and amino sugar and nucleotide sugar metabolism, alongside persistent hormone signaling [45]. This sustained metabolic activity, even after stress relief, may contribute to stress memory effects that prime the plant for future stress encounters. Notably, photosynthesis-related pathways were enriched specifically in LA and SP, indicating long-term adjustments of photosynthetic capacity during stress adaptation, with recovery of these pathways after stress relief [46].

The integration of miRNA and mRNA expression data revealed stage-specific regulatory networks with distinct functional compositions (Fig. 5; Table 1). In ER, miRNA-targeted genes were predominantly enriched in signal perception and transduction, including leucine-rich repeat (LRR) domain-containing proteins and protein kinases, as well as components involved in DNA replication protection such as the SLD5 subunit of the GINS complex [47]. This network configuration suggests that the initial response to salt stress involves rapid activation of signaling cascades coupled with safeguarding of genomic integrity—a critical priority when cells face stress-induced DNA damage. The ptc-miR6476-GINS circuit exemplifies this coordination, representing a novel link between miRNA regulation and DNA replication protection under stress.

In LA and SP, the networks expanded to include genes associated with sulfate assimilation and redox homeostasis, such as ATP sulfurylase, sulfate transporters, and glutaredoxins. The ptc-miR395-APS1 circuit, active during these phases, modulates sulfate assimilation and glutathione biosynthesis, directly linking to the enrichment of cysteine and methionine metabolism and glutathione metabolism pathways observed in KEGG analysis [48]. This coordination between miRNA regulation and metabolic pathway activity illustrates how post-transcriptional control supports sustained antioxidant defense during prolonged stress [10].

The RP network exhibited the greatest functional diversity, encompassing protein degradation pathways (ubiquitin-activating enzyme E1), nucleotide homeostasis regulators (MazG), transcription factors (MYB), and signaling proteins (LRR-containing proteins). The ptc-miR6457b-MazG circuit [49], which releases inhibition of MazG upon miR6457b down-regulation, may facilitate nucleotide pool homeostasis during recovery—a critical requirement for DNA repair and cellular resetting. The ptc-miR319-MYB module [50], with down-regulation of both miRNA and its target, points to coordinated inhibition of growth-related programs to prioritize recovery resources, consistent with the concept of a growth-defense switch.

The experimental design distinguishing Recovery Process (RP, TRvsT1 and TRvsT4) from Recovered State (RS, TRvsCK) allowed us to explore the concepts of “stress memory” versus “physiological resetting”. Genes and miRNA-mRNA modules active specifically in RP represent components actively involved in resetting cellular homeostasis, including the ptc-miR6457b-MazG and ptc-miR319-MYB circuits [51]. In contrast, modules that remained differentially expressed in RS—such as persistent ptc-miR395-APS1 activity and sustained enrichment of cysteine and methionine metabolism, biosynthesis of various secondary metabolites, and plant hormone signal transduction—may represent molecular stress memory that primes the plant for future stress encounters [16, 40]. The retention of 17 DEMs and 2,239 DEGs in RS, with 698 phase-specific genes, supports this interpretation.

Beyond well-conserved miRNA families, our analysis revealed several novel miRNAs with intriguing phase-specific expression patterns that may contribute to *P. yunnanensis*-specific adaptation strategies (Additional file 1: Table S15). novel0183, which was down-regulated during LA but up-regulated during RP, targets a serine/threonine protein kinase involved in stress signaling. This dynamic regulation suggests a role in fine-tuning signal transduction: suppression during prolonged stress may prevent over-activation of stress responses that could be detrimental to cellular homeostasis, while re-activation during recovery could help reset signaling pathways to pre-stress conditions, acting as a molecular switch in the transition from stress adaptation to physiological resetting. novel0128, which targets a protein kinase, showed sustained activity during RP and RS, potentially contributing to long-term signaling adjustments. Unlike conserved miRNA families that mediate core stress responses across plant species, these novel miRNAs may reflect species-specific regulatory innovations that fine-tune *P. yunnanensis*’ adaptation to its native habitat.

While the anti-correlated expression patterns and sequence complementarity provide strong computational evidence for the predicted interactions, direct experimental validation (e.g., RLM-RACE or dual-luciferase reporter assays) is required to definitively confirm miRNA-mediated cleavage and elucidate biological functions. Key candidates for such future studies include the ptc-miR395-APS1 module, the ptc-miR319-MYB module, and novel miRNAs such as novel0183 and novel0128. Translating these predictive networks into functionally validated regulatory mechanisms represents an important direction for our continued research.

## Conclusion

This study elucidates the dynamic, phase-specific post-transcriptional regulatory networks underlying salt stress adaptation in *P. yunnanensis*. By distinguishing five biologically defined phases—Early Response (ER), Long-term Adaptation (LA), Stress Progression (SP), Recovery Process (RP), and Recovered State (RS)—we uncovered how distinct miRNA-mRNA modules drive specific physiological transitions: from initial signal perception and DNA protection (ptc-miR6476-GINS), through sustained metabolic adaptation and antioxidant defense (ptc-miR395-APS1), to active recovery mechanisms (ptc-miR6457b-MazG, ptc-miR319-MYB) and the establishment of stress memory. Novel miRNAs such as novel0183 and novel0128 exhibited phase-specific dynamics, suggesting roles in fine-tuning stress signaling unique to *P. yunnanensis*. These findings provide a comprehensive predictive framework and valuable genetic resources for breeding stress-resilient trees.

## Supplementary Information

Additional file 1) Table S1. Statistics of clean reads from small RNA sequencing of P. yunnanensis leaves under control and salt-stress conditions. Table S2. Statistics of reads from mRNA sequencing of P. yunnanensis leaves under control (CK) and three salt-stress points (T1, T4, TR). Table S3. Primer sequences used for quantitative reverse transcription polymerase chain reaction (qRT-PCR) validation. Table S4. Families and expression levels of all 339 identified miRNAs across the salt treatments. Table S5. Sequences (mature and precursor) and expression levels (TPM) of the novel miRNAs predicted from the 12 small RNA libraries. Table S6. Expression levels (TPM) of all identified miRNAs in the four treatment points. Table S7. Statistics of differentially expressed miRNAs (DEMs) for each of the six pairwise comparisons among treatments. Table S8. Cluster assignment and expression data for DEMs presented in the heatmap. Table S9. Statistics of DEMs specific to five salt-stress stages (ER, LA, RS, SP, RP). Table S10. List of differentially expressed genes (DEGs) and their expression levels for the six pairwise comparisons. Table S11. Cluster assignment and expression data for DEGs presented in the heatmap. Table S12. Gene Ontology (GO) enrichment analysis results for each cluster of DEGs. Table S13. List of DEGs specific to the five salt-stress stages (ER, LA, RS, SP, RP). Table S14. Kyoto Encyclopedia of Genes and Genomes (KEGG) pathway enrichment analysis results for DEGs in the five salt-stress stages. Table S15. Predicted mRNA targets for the DEMs identified in the five stages. Table S16. Predicted binding sites and sequence complementarity of key miRNA-mRNA pairs.

Additional file 2) Figure. S1 Venn diagrams showing the overlap of (**a**) identified miRNAs and (**b**) expressed mRNAs across the four treatment points. Figure. S2 Principal component analysis (PCA) of mRNA expression profiles from four treatment points (CK, T1, T4, TR). Each point represents a biological replicate. Figure. S3 qRT-PCR validation of the expression patterns of (**a**) selected miRNAs and (**b**) three corresponding target mRNAs

## Data Availability

The raw sequencing data (miRNA-seq and RNA-seq) generated in this study have been deposited in the National Genomics Data Center (NGDC) (https://ngdc.cncb.ac.cn/bioproject) under the BioProject accession number PRJCA052778. The data will be publicly available upon publication.
