# Supplementary material for: Integrated miRNA-seq and RNA-seq analysis reveals stage-specific miRNA-mRNA regulatory networks in Populus yunnanensis under salt stress
Source: BMC Genomics. 2026 Apr 27;27:531. doi: 10.1186/s12864-026-12882-w (PMC13251124; doi:10.1186/s12864-026-12882-w)
Supplement: Supplementary file 2 — Additional file 2) Figure. S1 Venn diagrams showing the overlap of (a) identified miRNAs and (b) expressed mRNAs across the four treatment points. Figure. S2 Principal component analysis (PCA) of mRNA expression profiles from four treatment points (CK, T1, T4, TR). Each point represents a biological replicate. Figure. S3 qRT-PCR validation of the expression patterns of (a) selected miRNAs and (b) three corresponding target mRNAs [file 12864_2026_12882_MOESM2_ESM.docx]

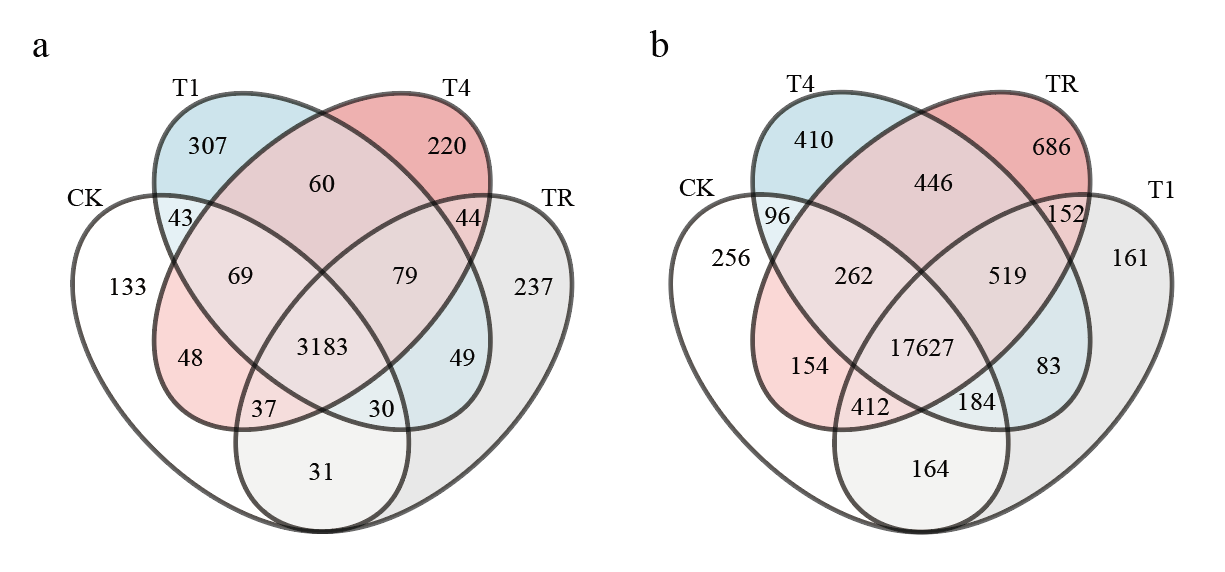


**Figure. S1** Venn diagrams showing the overlap of (a) identified miRNAs and (b) expressed mRNAs across the four treatment points.


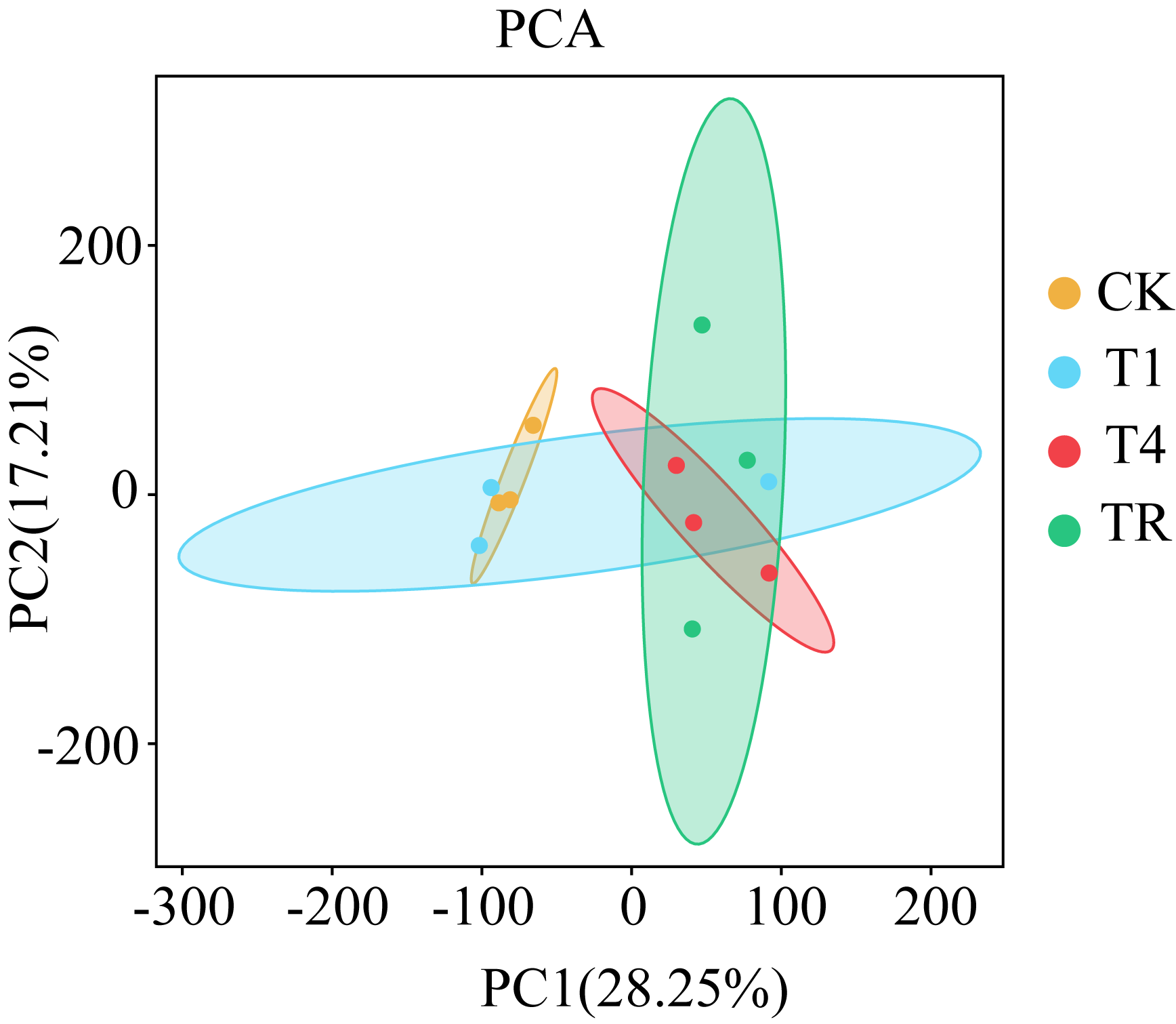


**Figure. S2** Principal component analysis (PCA) of mRNA expression profiles from four treatment points (CK, T1, T4, TR). Each point represents a biological replicate.


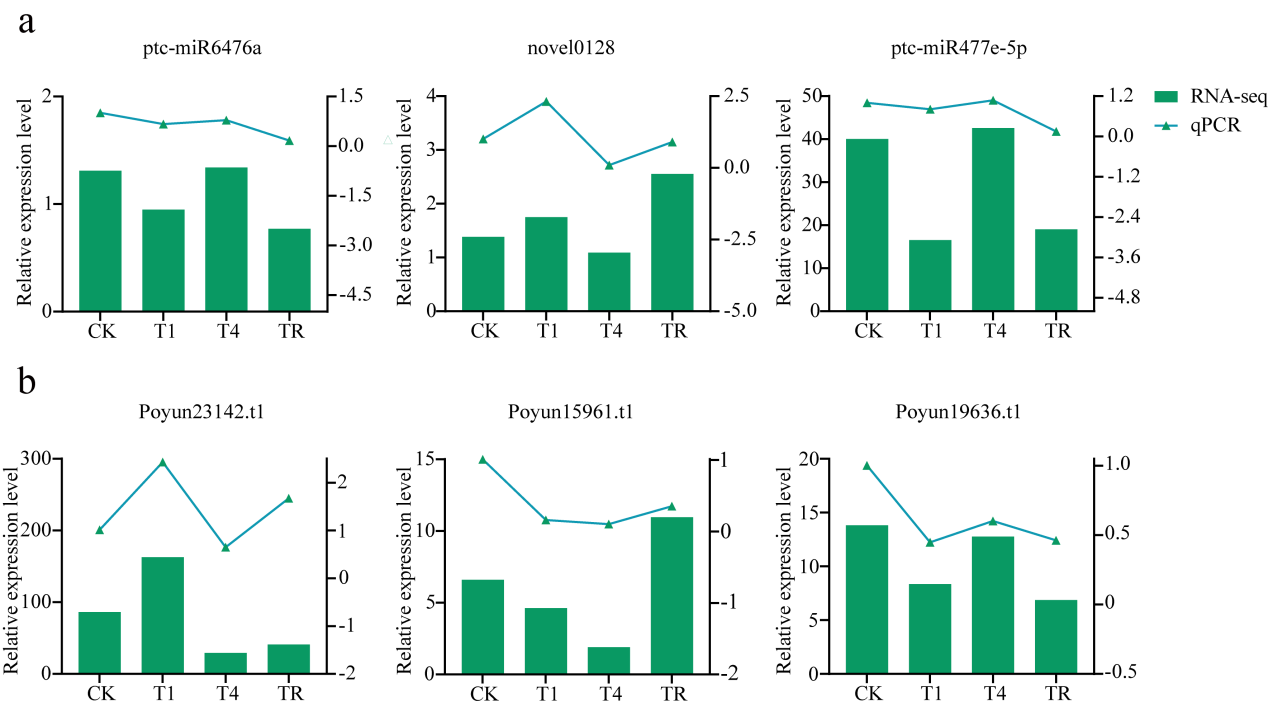


**Figure. S3** qRT-PCR validation of the expression patterns of (a) selected miRNAs and (b) three corresponding target mRNAs.
